# Community Structure of Lithotrophically-Driven Hydrothermal Microbial Mats from the Mariana Arc and Back-Arc

**DOI:** 10.3389/fmicb.2017.01578

**Published:** 2017-08-28

**Authors:** Kevin W. Hager, Heather Fullerton, David A. Butterfield, Craig L. Moyer

**Affiliations:** ^1^Department of Biology, Western Washington University Bellingham, WA, United States; ^2^National Oceanic and Atmospheric Administration Pacific Marine Environmental Lab, Joint Institute for the Study of the Atmosphere and Ocean, University of Washington Seattle, WA, United States

**Keywords:** Mariana Arc and back-arc, hydrothermal vents, community structure, microbial mats, ecosystem engineers

## Abstract

The Mariana region exhibits a rich array of hydrothermal venting conditions in a complex geological setting, which provides a natural laboratory to study the influence of local environmental conditions on microbial community structure as well as large-scale patterns in microbial biogeography. We used high-throughput amplicon sequencing of the bacterial small subunit (SSU) rRNA gene from 22 microbial mats collected from four hydrothermally active locations along the Mariana Arc and back-arc to explore the structure of lithotrophically-based microbial mat communities. The vent effluent was classified as iron- or sulfur-rich corresponding with two distinct community types, dominated by either Zetaproteobacteria or Epsilonproteobacteria, respectively. The Zetaproteobacterial-based communities had the highest richness and diversity, which supports the hypothesis that Zetaproteobacteria function as ecosystem engineers creating a physical habitat within a chemical environment promoting enhanced microbial diversity. Gammaproteobacteria were also high in abundance within the iron-dominated mats and some likely contribute to primary production. In addition, we also compare sampling scale, showing that bulk sampling of microbial mats yields higher diversity than micro-scale sampling. We present a comprehensive analysis and offer new insights into the community structure and diversity of lithotrophically-driven microbial mats from a hydrothermal region associated with high microbial biodiversity. Our study indicates an important functional role of for the Zetaproteobacteria altering the mat habitat and enhancing community interactions and complexity.

## Introduction

Recognizing and cataloging the microbial biodiversity at extant hydrothermal vents is critical to gain a better understanding of current and ancient ecosystem functions and how the taxa present play a role in global geochemical processes (Gilbert et al., 2011; Reed et al., 2014). The steep redox gradients and high concentration of reduced substrates [e.g., Fe(II), H_2_S, and H_2_] in hydrothermal vent habitats provide energetically favorable conditions that support luxuriant microbial mats with phylogenetically diverse lithoautotrophic microbes (Emerson and Moyer, 2010; Amend et al., 2011). This spectrum of geochemistry is thought to be similar to that of early Earth and as such, hydrothermal vents are a compelling system to study early life on Earth and may provide insights into other potentially habitable zones such as Saturn's moon, Enceladus (Martin et al., 2008; McKay et al., 2008). Early Earth may have been drastically modified by iron-oxidizing bacteria as they are thought to have been partially, if not fully, responsible for the global pattern of banded iron formations deposited during the Precambrian (Konhauser et al., 2002; Chan et al., 2016a). Iron is the second most abundant metal in Earth's crust (Kappler et al., 2015), and it represents a large and ancient energy source for iron-oxidizing bacteria (Planavsky et al., 2009). Therefore, hydrothermal vent systems allow for investigations into the fundamentals of microbial ecology and biogeography as well as planetary processes such as global carbon and mineral cycling (Nakagawa and Takai, 2008; Dick et al., 2013; Resing et al., 2015).

The hydrothermally active regions of the Mariana Arc and back-arc systems are formed by differential volcanic activity from the subduction and melting of the Pacific plate beneath the Philippine plate (Fryer, 1996). Relative to mid-ocean ridge hydrothermal systems, the geochemistry of the hydrothermal vent effluent (e.g., concentrations of reduced metals, H_2_, H_2_S, CO_2_, and ${\text{NH}}_{4}^{+}$) across the Mariana region is highly heterogeneous due to the wide range in magmatic volatile content and magma chemistry of the Island Arc and back-arc. Fluid chemistry may be dominated by magmatic CO_2_, as at NW Eifuku (Lupton et al., 2006, 2008), or by active volcanism and magmatic SO_2_, as at NW Rota-1 (Butterfield et al., 2011), or may be more rock-buffered as at Urashima and Snail sites on the back-arc (Nakamura et al., 2013; Ishibashi et al., 2015). Significant variation in fluid chemistry within a vent field on a single submarine volcano is not uncommon, due to sub-seafloor reaction between host rock and fluids enriched in magmatic gases (e.g., Champagne and Yellow Cone vents from NW Eifuku). This high variability in vent effluent geochemistry compounded over the expansive geographic area of the Mariana region harbors disparate habitats that offer the opportunity for niche differentiation, a wide range of metabolic potential, and diverse microbial communities (Davis and Moyer, 2008).

Hydrothermal vent microbial community structure in the Mariana region was shown to be extremely diverse and has been split into three groups dominated either by Zetaproteobacteria, Epsilonproteobacteria, or putative heterotrophic phylotypes (Davis and Moyer, 2008). The Zetaproteobacteria are a more recently described class of iron-oxidizing bacteria (Emerson et al., 2007), and a complex picture of biogeography has begun to emerge as they are found globally in an array of iron-rich environments including hydrothermal vent microbial mats from the South Tonga Arc; Iwo-Jima, Japan; Tutum Bay, Papua New Guinea; and the Mid Atlantic Ridge (Forget et al., 2010; Meyer-Dombard et al., 2013; Hoshino et al., 2015; Scott et al., 2015). Zetaproteobacteria have also been detected in hydrothermal borehole fluids (Kato et al., 2009b), continental subsurface water (Emerson et al., 2015), near shore estuaries (McBeth et al., 2013), estuarine oxygen minimum zones (Field et al., 2016), and non-venting deep continental margins (Rubin-Blum et al., 2014). Recent studies hypothesize that Zetaproteobacteria are microbial ecosystem engineers because they have the genetic potential for the production of organic carbon and the capacity to shape the environment by producing iron oxyhydroxide minerals and exopolysaccharides, which in turn provide structure to the mats and can alter the local geochemistry, enhancing microbial diversity (Forget et al., 2010; Fleming et al., 2013; Meyer-Dombard et al., 2013; Jesser et al., 2015; Chan et al., 2016b). Due to the variability of the vent effluent composition, the Mariana region also has sulfur-rich hydrothermal habitats. Sulfur- and hydrogen-oxidizing Epsilonproteobacteria have been detected in microbial mats and hydrothermal fluids as the primary lithotrophic drivers at these locations (Davis and Moyer, 2008; Huber et al., 2010; Meyer and Huber, 2014).

In addition to the observed geographic-scale variation across vent habitats, community structures can be can be examined on a spatial scale of millimeters within individual microbial mats. Sampling at smaller spatial scales has received much attention in studies of soils and photosynthetic mats (Fike et al., 2008; Harris et al., 2013; Raynaud and Nunan, 2014; Cordero and Datta, 2016), but only recently has high-resolution sampling of deep-sea hydrothermal mats been highlighted (Breier et al., 2012; Teske et al., 2016). Unlike easily accessible photosynthetic mats, microbial mats in the deep sea are difficult to study on a fine scale due to the limitations of sample collection with remotely operated vehicles. Systematic, fine-scale sampling of iron mats from Lō'ihi Seamount, Hawai'i has revealed different abundances of functional genes and extracellular structures from visibly different mat morphologies (Fleming et al., 2013; Jesser et al., 2015; Fullerton et al., 2017) that occur in microbial mats that can be over a meter deep (Edwards et al., 2011) exhibiting millimeter scale redox gradients, especially at their surface (Glazer and Rouxel, 2009). Further, microscopy studies of iron-oxidizing bacteria reveal that cells are not evenly distributed in mats, but rather they develop into actively growing fronts (Chan et al., 2016b) that can oxidize iron at a rate of up to 52 μmole hr^−1^ and can accrete mat material at ~2.2 cm yr^−1^ (Emerson et al., 2017). These data confirm the importance of addressing spatial heterogeneity through fine-scale sampling of hydrothermal microbial mats.

The Mariana Arc and back-arc hydrothermal vent microbial communities have been described with high microbial biodiversity using small subunit (SSU) rRNA gene clone libraries and community fingerprinting analyses (Davis and Moyer, 2008). For this study, we used high-throughput, second generation SSU rRNA gene amplicon sequencing (Caporaso et al., 2012; Pedrós-Alió, 2012) in an effort to comprehensively investigate and better understand the community structure of microbial mats along the Mariana Arc and back-arc. We further expand on the importance of sampling scale by juxtaposing the microbial diversity of mats collected with a fine-scale sampling device (e.g., biomat sampler) to more commonly used scoop samplers. These results provide novel insights into patterns of biogeography, ecology, and microbial biodiversity of the lithotrophic drivers of these hydrothermal communities.

## Methodology

### Sample collection

**Figure 1:**
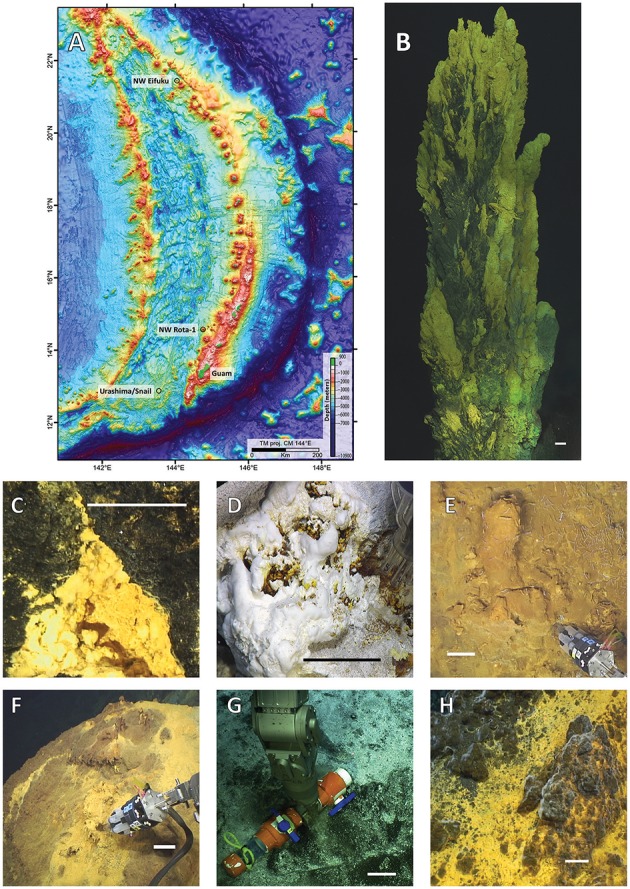
**(A)** Bathymetric map of the Mariana region with black circles designating dive locations. Representative photos of mat collection sites: **(B)** Golden Horn chimney at Urashima; **(C)** Marker 104 at Snail Vents; **(D)** Champagne Vents; **(E)** Yellow Cone Mkr 146, and **(F)** Yellow Cone Marker 124 at NW Eifuku; **(G)** Iceberg and **(H)** Olde Iron Slides at NW Rota-1. Images **(F,G)** include the biomat syringe sampler and an RNA*later* Scoop (LSc), respectively. Scale bars represent 10 cm. Map is courtesy of Susan Merle, NOAA EOI/OSU.

Microbial mats were collected at the Mariana back-arc sites Snail (also known as the Fryer Site) and Urashima and at the Mariana Arc sites NW Eifuku and NW Rota-1 (Figure 1A) during R/V *Roger Revelle* cruise 1413 (11/29/2014–12/21/2014) with remotely operated vehicle *Jason II*. A total of 22 samples were collected with either the biomat syringe sampler (Breier et al., 2012; Figure 1F) or scoop sampler (Figure 1G, Table 1). Scoop samples were preserved in RNA*later* (ThermoFisher Scientific, Waltham, MA) at depth (LSc), or RNA*later* was added after the scoop was brought to the surface (Sc). All mats collected were stored at −80°C upon processing until DNA extractions. Sample names consist of *Jason II* dive number (797–801) followed by biomat sampler cassette letter and individual syringe number(s) or the type of scoop and the scoop number (e.g., biomat sampler: 797B3 and scoop sampler: 797LSc1). Mats from three locations were collected with both biomat and scoop samplers to compare community structure and diversity in relation to sampling technique. Vent effluent was collected with the Hydrothermal Fluid and Particle Sampler (Butterfield et al., 2004) and analyzed on board for total hydrogen sulfide, pH, and dissolved hydrogen. Iron was measured by atomic absorption at Pacific Marine Environmental Lab in Seattle, WA.

**Table 1:** Sample location details and general descriptions of microbial mats.

**Sample**	**Vent field**	**Site**	**Latitude**	**Longitude**	**Depth (m)**	**Description**
797D156	Snail	Mkr 108	12° 57.166′ N	143° 37.142′ E	2,850	Iron mat with black Mn outer surface
797D234	Snail	Mkr 108	12° 57.166′ N	143° 37.142′ E	2,850	Iron mat with black Mn outer surface
797B12	Urashima	Snap Snap	12° 55.333′ N	143° 38.950′ E	2,928	Flocculant iron mat
797B3	Urashima	Snap Snap	12° 55.333′ N	143° 38.950′ E	2,928	Flocculant iron mat
797B56	Urashima	Snap Snap	12° 55.333′ N	143° 38.950′ E	2,928	Flocculant iron mat
797LSc1	Urashima	Snap Snap	12° 55.333′ N	143° 38.950′ E	2,928	Flocculant iron mat
797C34	Urashima	Saipanda Horn	12° 55.333′ N	143° 38.950′ E	2,928	Flocculant iron mat
801Sc8	Urashima	Golden Horn (base)	12° 55.340′ N	143° 38.957′ E	2,930	Flocculant iron mat
801X126	Urashima	Golden Horn (base)	12° 55.343′ N	143° 38.953′ E	2,931	Flocculant iron mat
801X345	Urashima	Golden Horn (middle)	12° 55.343′ N	143° 38.953′ E	2,928	Flocculant iron mat
801LSc4	Urashima	Golden Horn (top)	12° 55.343′ N	143° 38.953′ E	2,922	Flocculant iron mat
798B123456	NW Eifuku	Champagne	21° 29.244′ N	144° 2.485′ E	1,606	Creamy white sulfur mat at vent orifice
798LSc3	NW Eifuku	Yellow Cone (Mkr 124)	21° 29.274′ N	144° 2.519′ E	1,584	Flocculant iron mat
798C346	NW Eifuku	Yellow Cone (Mkr 146)	21° 29.265′ N	144° 2.519′ E	1,579	Flocculant iron mat
798LSc1	NW Eifuku	Yellow Cone (Mkr 146)	21° 29.265′ N	144° 2.519′ E	1,579	Flocculant iron mat
799B156	NW Eifuku	Yellow Cone (Mkr 146)	21° 29.264′ N	144° 2.524′ E	1,581	Flocculant iron mat
799D124	NW Eifuku	Yellow Cone (Mkr 146)	21° 29.264′ N	144° 2.524′ E	1,580	Flocculant iron mat
799D3	NW Eifuku	Yellow Cone (Mkr 146)	21° 29.264′ N	144° 2.524′ E	1,581	Flocculant iron mat
799D56	NW Eifuku	Yellow Cone (Mkr 146)	21° 29.264′ N	144° 2.524′ E	1,581	Flocculant iron mat
800LSc2	NW Rota-1	Iceberg	14° 36.061′ N	144° 46.577′ E	527	Thin white mat on volcanic sand
800B12456	NW Rota-1	Olde Iron Slides	14° 36.056′ N	144° 46.656′ E	567	Iron tufts adjacent to white filamentous mat
800Sc8	NW Rota-1	Olde Iron Slides	14° 36.056′ N	144° 46.656′ E	567	Iron tufts mixed with white filamentous mat

### DNA extractions, amplification, and sequencing

For each sample, three to five extractions using ~0.5 g of mat material for each extraction were done using the FastDNA SPIN Kit for Soil (MP Biomedicals, Santa Ana, CA) after removal of excess fluid (sea water or RNA*later)*. The manufacture's protocol was followed with the following modifications: 250 μl 0.5 M sodium citrate pH 5.8 was added in place of 250 μl of the sodium phosphate buffer. Lysis was performed with two rounds of bead beating for 45 s at a setting of 5.5 using the FastPrep instrument (MP Biomedicals) with samples being placed on ice between runs. DNA was eluted in 100 μl 1.0 mM Tris pH 8.0. Genomic DNA was quantified with a Qubit 2.0 fluorometer using the dsDNA high sensitivity kit (ThermoFisher Scientific).

The V3-V4 variable regions of the SSU rRNA gene were amplified via polymerase chain reaction (PCR) from all mat samples using bacterial primers 340F (5′-CCTACGGGNGGC WGCAG-3′) and 784R (5′-GGACTACHVGGGTATCTAATCC-3′) according to Klindworth et al. (2012) with overhang sequences on the 5′ ends (not shown) compatible with Illumina adapters. Triplicate PCRs were performed in 25 μl reactions with 2X KAPA HiFi HotStart ReadyMix (Kapa Biosystems, Wilmington, MA), 0.1 mM forward/reverse primers, and 25 ng template DNA. The following PCR conditions were used: 3 min at 95°C; 25 cycles of 30 s at 95°C, 30 s at 55°C, and 30 s at 72°C; and a final elongation of 5 min at 72°C. PCR products were pooled and purified using Agencourt AMPure XP beads (Beckman Coulter, Brea, CA). Illumina Nextera XT (Illumina Inc.) adapters with unique index combinations were added to each sample in a 50 μl PCR using 2X KAPA HiFi HotStart ReadyMix with the following conditions: 3 min at 95°C; 8 cycles of 30 s at 95°C, 30 s at 55°C, and 30 s at 72°C; and a final elongation of 5 min at 72°C. Products were again purified with Agencourt AMPure XP beads. Libraries were then quantified with a Qubit 2.0 fluorometer and size validated with a 2100 Bioanalyzer (Agilent Technologies, Santa Clara, CA). Sequencing was performed on an Illumina MiSeq as per manufacturer's protocol generating 2 × 300 bp paired-end reads. Sequence data are available through the NCBI Sequence Read Archive study number SRP092903 (BioProject: PRJNA352433).

### Sequence processing

Reads in which 75% of the base calls had a QScores below 30 were discarded from further analysis using FASTQ Quality Filter of the FASTX-Toolkit (http://hannonlab.cshl.edu/fastx_toolkit/). Remaining sequences were processed using the mothur software package as previously described (Schloss et al., 2009; Kozich et al., 2013). Briefly, after forming contigs from the paired-end reads, PCR primers were trimmed off and any sequence with a homopolymer >8 bases or any sequences with ambiguous base calls were eliminated from further processing. Reads were pre-cluster with the “pre.cluster” command with a threshold of four-nucleotide differences. Chimeras were removed with UCHIME (Edgar et al., 2011). Sequences were binned into operational taxonomic units (OTUs) based on 97% sequence similarity using “cluster.split”, and OTUs were classified to the genus level using RDP training set v.9 (Wang et al., 2007; Cole et al., 2013).

The program ZetaHunter (https://github.com/mooreryan/ZetaHunter) assigns sequences classified as Zetaproteobacteria to previously defined Zetaproteobacterial OTUs (zOTUs) (Schloss et al., 2009; McAllister et al., 2011; Quast et al., 2012). The most abundant read from each OTU classified as Zetaproteobacteria was assessed using ZetaHunter to further classify these OTUs for comparison to previous studies.

### Diversity and statistical analyses

OTU bins at the level of 97% sequence similarity as determined with mothur were used in all downstream analyses. We subsampled to the number of reads in the least sequenced sample (54,803 contigs) for calculation of diversity indices with the “summary.single” command. A distance matrix was calculated (Yue and Clayton, 2005) and a community structure dendrogram was constructed in BioNumerics v.7.5 (Applied Maths, Sint-Martens-Latem, Belgium) using unweighted pair group method with arithmetic mean (UPGMA) including calculation of cophenetic correlations. Rarefaction curves were calculated using mothur based on the number of observed OTUs per sample and 1,000 iterations. Abundant OTUs were determined by selecting OTUs with >1% of the total reads/sample in at least one sample. The vegan and gplots packages in R were used (R Core Team, 2014; Oksanen et al., 2015; Warnes et al., 2015) to visualize abundant OTUs belonging to the Zeta-, Epsilon-, and Gamma-proteobacteria with double hierarchical clustering based off Bray-Curtis dissimilatory matrices. Metastats is an application that uses count data from multiple populations to determine which OTUs are significantly different between populations (White et al., 2009). Implemented through mothur, a Metastats analysis was used to determine which of these OTUs had a significantly different abundance (*p* < 0.05) between groups established by clustering hierarchy. To link community structure to select environmental factors we performed a canonical correspondence analysis (CCA) with the vegan package in R using proportions of reads for the abundant Zeta-, Epsilon-, and Gamma-proteobacterial OTUs and environmental concentrations for Fe, H_2_S, and H_2_ (Ter Braak, 1986). Analysis of variance (ANOVA) was used to test for statistical significance (*p* < 0.05) of the CCA.

## Results

### Site descriptions

Microbial mats were collected from four active hydrothermal fields from both the Mariana Arc and back-arc. Sample collection locations and images depicting representative microbial mats are shown in Figure 1, and the general features of mats collected are described in Table 1. Snail, an on-axis back-arc site, was characterized by thick orange mats with a black surface likely composed of manganese (Figure 1C). Urashima is an off-axis, back-arc field ~5 km to the southeast of Snail characterized by tall iron-sulfide chimney structures layered with flocculent orange microbial mats. Golden Horn Chimney was the largest of these structures reaching 13 m high (Figure 1B). The arc seamount NW Eifuku included creamy, white mats near liquid CO_2_ bubbling at Champagne Vent (Figure 1D) as well as yellow to orange mats located at Yellow Cone Markers 146 and 124 (Figures 1E,F). Yellow Cone had small, chimney-like structures formed from flocculent mat material, but no large mineralized chimneys such as Golden Horn were observed. The seamount NW Rota-1 was primarily dominated by thin white microbial mats comparable to those collected at Iceberg (Figure 1G); however, Olde Iron Slides had thin tufts of yellow-orange mat as well (Figure 1H). The fluids collected adjacent to microbial mats from Champagne and Iceberg are referred to as sulfur-dominated; they had H_2_S to Fe molar ratio values of >4,000x with respect to fluids collected amidst the flocculent, yellow to orange microbial mats, which were high in Fe and considered iron-dominated (Table 2). Fluids from Champagne also had a relatively high concentration of H_2_ compared to all other locations in this study. No fluid for geochemical analyses were collected at Olde Iron Slides.

**Table 2:** Geochemical profiles for mat collection sites.

**Vent field**	**Site**	**Tmax (°C)**	**pH**	**Fe (μM)**	**H_2_S (μM)**	**H_2_ (μM)**
Snail	Mkr 108	36.0	6.7	19.3	<0.4	<0.01
Urashima	Saipanda Horn/Snap Snap	18.7	6.5	48.5	<0.4	0.01
Urashima	Golden Horn (base)	10.0	6.6	54.0	<0.4	0.03
Urashima	Golden Horn (middle)	10.3	6.5	67.0	<0.4	0.02
Urashima	Golden Horn (top)	15.9	6.3	66.1	0.4	0.01
Urashima	Mid-water background	2.0	7.5	15.3	<0.4	0.02
NW-Eifuku	Champagne (Mkr144)	63.0	4.9	14.7	2758.1	0.70
NW-Eifuku	Yellow Cone (Mkr124)	22.5	5.6	221.3	0.8	<0.01
NW-Eifuku	Yellow Cone (Mkr146)	30.5	5.7	96.7	4.0	0.02
NW-Eifuku	Eifuku background plume	2.0	7.5	0.1	0.4	<0.01
NW Rota-1	Menagerie (near Iceberg)	19.1	5.4	<0.1	816.0	<0.01

### Sequencing and community structure

A total of 22 samples of microbial mats were collected and analyzed including 20 from locations with iron-dominated fluids including Snail, Urashima, NW Eifuku, and NW Rota-1. One mat sample was collected from Champagne and Iceberg respectively, where fluids were sulfur-dominated. In total 17,515,651 raw, paired-end sequences with an average length of 420 bp covering the V3-V4 regions of the SSU rRNA gene were generated. After quality filtering with mothur, a total of 6,770,752 contigs remained. At a 97% similarity cutoff 62,520 OTUs were generated of which 162 were abundant with >1% of the total reads in at least one sample. Of these OTUs, were 30 cosmopolitan taxa found across all iron-dominated mats.

**Figure 2:**
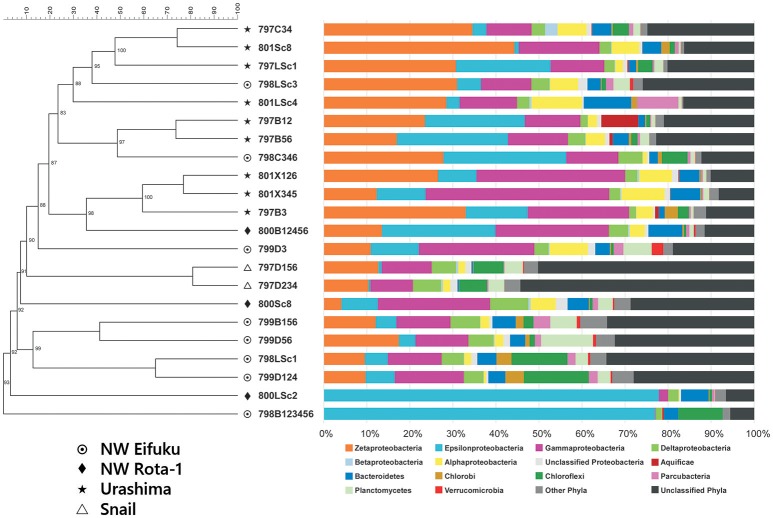
OTU-based dendrogram with cophenetic correlation values at nodes and a scale bar representing percent similarity. Stacked bar graphs of community structure including Proteobacteria at the class level and other abundant phyla for 22 microbial mats from the Mariana Arc and back-arc.

As seen in Figure 2, at the phylum level, all samples had a large abundance of the Proteobacteria with a majority of these reads belonging to the classes Zetaproteobacteria, Epsilonproteobacteria, and Gammaproteobacteria. Unclassified bacteria also made up a large portion of all communities. Both mats from Snail (797D156 and 797D234) contained >50% unclassified bacteria whereas the average and standard deviation for all other samples was 18.8 ± 9.5%. Other phyla found in notable abundance include Chloroflexi, Bacteroidetes, and Planctomycetes.

**Figure 3:**
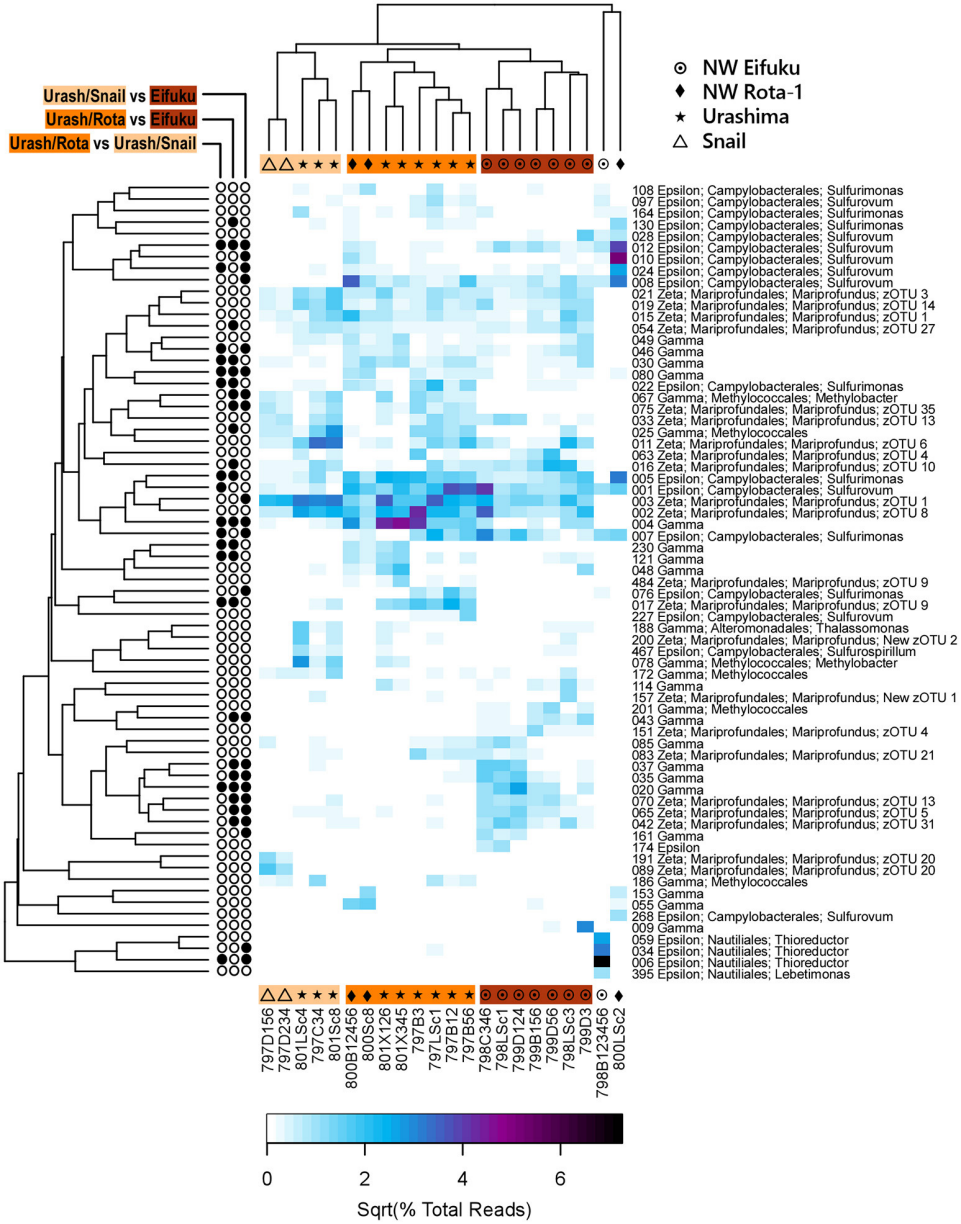
Heatmap of Zeta-, Epsilon-, and Gamma-Proteobacterial OTUs with a maximum abundance >1% of reads in at least one sample. OTU number, class, family, and genus are displayed where sequences are classifiable. The zOTU classification is provided for the Zetaproteobacteria. Square root transformed values of the percentage of total reads per sample are plotted. Three groupings of iron-dominated microbial mat communities are present: an Urashima/Snail group (Urash/Snail), an Urashima/NW Rota-1 group (Urash/Rota), and a NW Eifuku group (Eifuku) are indicated with light, medium, and dark orange shading, respectively. Filled circles in the three columns left of the heatmap indicate a significant difference (*p* < 0.05) in OTU abundance with respect to pairwise comparisons between the three groups of iron-dominated mats using Metastats.

The mat communities from iron-dominated fluids clustered together with the exclusion of the two mat communities from sulfur-dominated fluids (Figure 2). These two sulfur-dominated communities also had low similarity to each other as determined by the Yue-Clayton similarity measure and <25% of other taxa than the identified Epsilonproteobacteria, respectively, thereby comprising relative simple community structures. Cluster analysis revealed mixed clustering hierarchy among vent fields (Figure 2). However, when only comparing the 69 abundant Zeta-, Epsilon-, and Gamma-proteobacterial OTUs, the iron-rich mats formed three distinct clusters. One consisted of all the mats from NW Eifuku, and the other two were composed of Snail/Urashima mats and NW Rota-1/Urashima communities (Figure 3). A Metastats analysis revealed that 33 of these OTUs had a significant difference (*p* < 0.05) in abundance among the three groups (Figure 3).

Zetaproteobacterial phylotypes were detected in all flocculent, orange communities associated with iron-dominated fluids ranging from 4.1 to 44.3% (average ± standard deviation = 20.0 ± 10.9%) of the community composition. All Zetaproteobacterial OTUs were classified by RDP as the same genus, *Mariprofundus*, and the program ZetaHunter allowed for finer-scale OTU classification. All but two of the abundant Zetaproteobacterial OTUs (New zOTU 1 and 2) fit into previously characterized zOTUs. The two most abundant zOTUs (1 and 8) were found in all of the iron mat communities examined. Other zOTUs were endemic to one location; e.g., zOTU 20 was restricted to only Snail (Figure 3). Although detected (data not shown), no abundant OTUs were classified as zOTU 2 or 11. The two sulfur-dominated communities from Champagne and Iceberg had <0.06% of the reads classified as Zetaproteobacteria.

Epsilonproteobacteria were detected in every mat community, and the two sulfur-dominated microbial mats from Champagne and Iceberg had the highest percent of reads belonging to Epsilonproteobacteria at >77% (Figure 2). The Champagne mat was composed primarily of the genera *Thioreductor* and *Lebetimonas* (Figure 3). A single *Thioreductor* OTU represented nearly 53% of the total reads from the Champagne mat (Figure 3). The Iceberg microbial community was composed predominantly of the genera *Sulfurimonas* and *Sulfurovum* with 26% of the total reads assigned to a single Sulfurovum OTU (Figure 3). Epsilonproteobacteria were well represented in iron-dominated mats as well, where they comprised 0.4–28.5% (average ± standard deviation = 13.9 ± 9.3%) of the reads (Figure 2). These abundant Epsilonproteobacterial OTUs present were also of the genera *Sulfurovum* and *Sulfurimonas* (Figure 3).

Gammaproteobacteria made up a large fraction of the community in iron-dominated mats as well. The Gammaproteobacteria range from 5.5 to 42.7% (average ± standard deviation = 17.4 ± 9.2%) of the reads in iron-dominated microbial mats, but they were found in low abundance (≤2.1%) in the sulfur-dominated mats (Figure 2). Of the 25 abundant Gammaproteobacterial OTUs, six belonged to the order Methylococcales and 18 could not be classified past the class level. BLAST searches of these unclassified Gammaproteobacteria indicated close relation to other environmental samples from marine hydrothermal environments (Supplemental Table 1).

**Figure 4:**
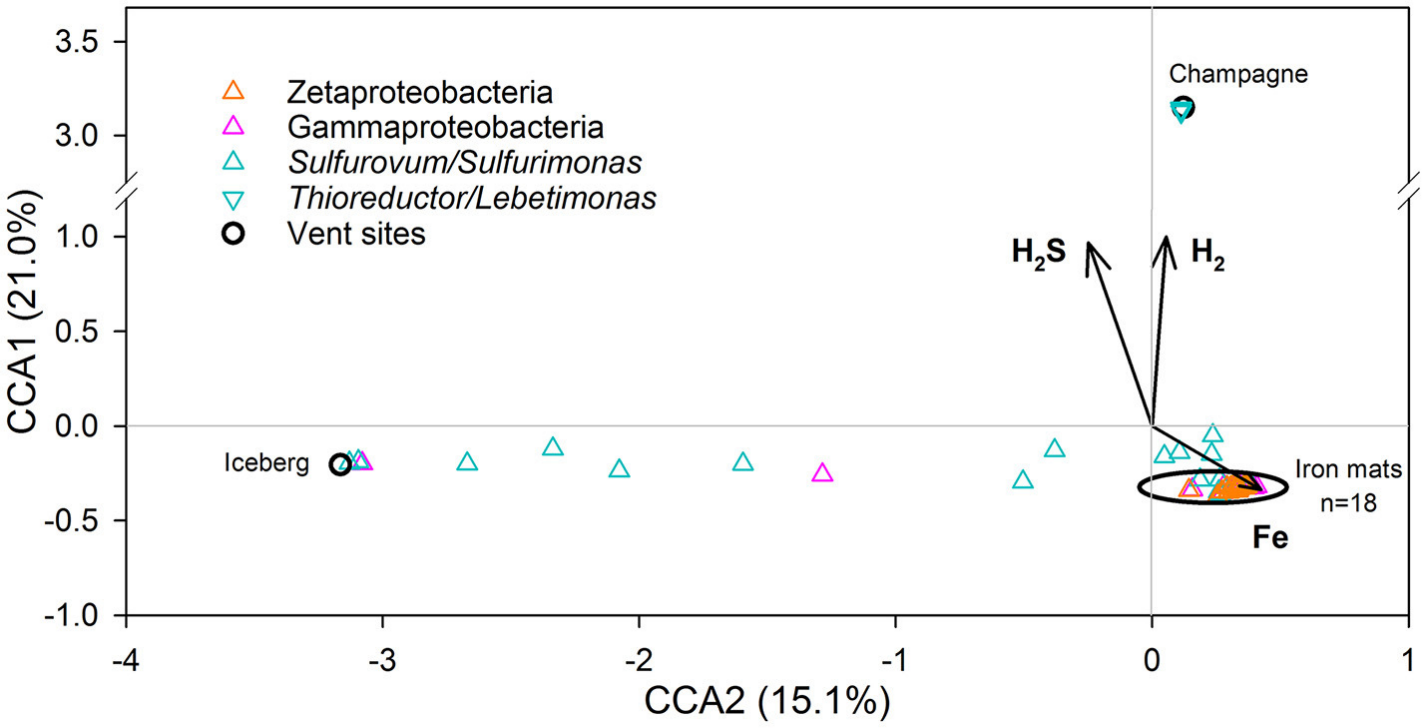
CCA triplot of community structure in relation to selected environmental variables for microbial mats from hydrothermal vents along the Mariana Arc and back-arc. Colored triangles represent abundant OTUs of the classes Zetaproteobacteria (*n* = 22) and Gammaproteobacteria (*n* = 25) and the genera *Sulfurovum*/*Sulfurimonas* (*n* = 18) and *Thioreductor*/*Lebetimonas* (inverted triangles, *n* = 4) of the Epsilonproteobacteria. Coordinates of mat collection sites are indicated with black circles. Percent of total variability explained by each axis is in parentheses on the axis label. The model is significant at *p* < 0.005.

ANOVA on the CCA with the abundant Zeta-, Epsilon-, and Gamma-proteobacterial OTUs and environmental parameters pertaining to inorganic electron donors (Fe, H_2_S, and H_2_) was significant (*p* = 0.005); 36.1% of the total variability in community structure was captured by CCA1 and CCA2 (Figure 4). When tested, the environmental parameters of temperature and pH were not found to be significant (data not shown). All locations with iron-dominated fluids formed a tight grouping overlapping with the Zetaproteobacterial OTUs and all but two of the Gammaproteobacterial OTUs. The Sulfurovum/Sulfurimonas OTUs formed a diffuse group including the two remaining Gammaproteobacterial OTUs. Champagne and the four abundant Thioreductor/Lebetimonas OTUs constituted a distinct third cluster.

### Diversity estimates

Calculation of alpha diversity metrics based on OTUs defined at 97% sequence similarity allow for comparisons among samples (Table 3). An average Good's coverage of 0.98 indicated sufficient sequencing depth. The range and average values for Chao1 richness estimator, non-parametric Shannon diversity, and inverse Simpson diversity metrics, respectively, were 1,200–8,220 (average ± standard deviation = 4,646 ± 1,673), 2.1–6.3 (average ± standard deviation = 5.0 ± 1.0), 3.3–145.4 (average ± standard deviation = 50.0 ± 40.0) as seen in Table 3. The iron-dominated mats all had greater richness, diversity, and observed number of OTUs when compared to the two sulfur-dominated mats.

**Table 3:** Sequencing information and diversity metrics.

**Sample**	**Number of sequences**	**Observed OTUs**	**Good's coverage**	**Chao-1 richness**	Shannon diversity^*^	**Inverse Simpson diversity**	**Shannon evenness**
797D156	307,131	3,524	0.97	6,208	6.3	145.4	0.76
797D234	176,609	3,409	0.97	5,922	6.3	140.6	0.76
797B12	206,039	1,814	0.98	3,249	4.7	28.7	0.62
797B3	366,647	1,500	0.99	3,182	4.0	13.8	0.54
797B56	210,604	2,252	0.98	3,938	5.2	42.0	0.67
797LSc1	582,358	2,644	0.98	5,028	5.2	34.8	0.65
797C34	94,770	2,209	0.98	3,585	5.2	35.1	0.67
801Sc8	125,894	1,915	0.98	3,725	4.5	26.1	0.59
801X126	54,806	2,545	0.98	4,298	4.6	17.1	0.58
801X345	63,308	2,905	0.97	8,220	4.7	17.8	0.58
801LSc4	114,835	1,404	0.99	3,060	3.8	17.1	0.51
798B123456	233,627	504	0.99	1,200	2.1	3.3	0.34
798LSc3	420,287	2,656	0.97	5,991	5.6	80.9	0.70
798C346	379,737	1,756	0.98	3,819	4.2	16.1	0.55
798LSc1	334,615	2,255	0.98	4,840	5.4	62.7	0.69
799B156	348,417	3,091	0.97	5,626	6.0	92.5	0.73
799D124	271,779	1,712	0.98	3,316	4.9	43.6	0.65
799D3	1,123,677	2,929	0.97	5,918	5.6	61.9	0.70
799D56	92,953	2,606	0.98	4,095	5.7	76.5	0.71
800LSc2	350,113	1,495	0.99	3,028	3.4	8.7	0.46
800B12456	361,680	3,056	0.97	5,813	5.3	33.1	0.64
800Sc8	374,997	4,466	0.96	8,155	6.3	101.9	0.74

*All metrics calculated with subsampling to the lowest sequencing effort, 54,806 sequences*.

* *Non-parametric*.

Mat communities collected using scoops showed higher diversity than associated with biomat samples. At three locations both scoops and biomat samples were taken in the same place: 797LSc1 corresponds with 797B12 and 797B56, 798LSc1 corresponds with 798C346, and 800Sc8 corresponds with 800B12456. All the scoop communities had higher values for observed OTUs, Chao1 richness, and non-parametric Shannon diversity than their corresponding biomat communities with the exception that 797B56 had the same value for the non-parametric Shannon metric as 797LSc1 (Table 3). Rarefaction analysis also revealed higher richness in the mats collected with scoops compared to the corresponding biomat samples in all cases (Supplemental Figure 1).

## Discussion

### Community structure comparisons

Two distinct community types were identified based on the prevalent taxa. The first of these are the Zetaproteobacterial-dominated communities that were present only in association with the iron-dominated vent effluent. The second community type lacked Zetaproteobacteria; however, they had high levels of Epsilonproteobacteria and were found only in association with the sulfur-dominated fluids. These sulfur-dominated communities were present only at the Mariana Arc sites Champagne, NW Eifuku and Iceberg, NW Rota-1. Both community types contain an abundance of bacteria related to known lithoautotrophs to drive community primary production, and no mats were composed primarily of putative heterotrophic taxa as seen previously (Davis and Moyer, 2008).

The majority of the microbial mats we examined were of the Zetaproteobacteria-dominated community type and exhibited a high level of variability among the remaining community members (Figure 2). These Zetaproteobacterial-dominated mats were collected from all four dive locations (Table 1), and their respective community structure does not appear to correlate with location when all taxa are included (Figure 2). Only 18.5% of the abundant OTUs were found across all iron-dominated mats, which can lend explanation to the low Yue-Clayton similarities and high beta diversity in community structure observed between mat communities (Figure 2).

Biogeographic patterns emerged only when the variation of less abundant classes and the unclassified OTUs were removed from cluster analysis. The Zeta-, Epsilon-, and Gamma-proteobacteria, often the lithotrophic drivers of hydrothermal microbial mats, were found in high abundance in the iron mats here, and are likely the most ecologically significant members in these communities (Figure 2). With only the abundant OTUs belonging to the Zeta-, Epsilon-, and Gamma-proteobacteria, the Zetaproteobacterial-dominated mats of NW Eifuku all clustered together in one group (Figure 3). The two other groups are composed of communities from Snail, Urashima, and NW Rota-1, which are all relatively close to one another whereas NW Eifuku is hundreds of km north of these three vent fields (Figure 1A). These data support previous research showing community structure variability corresponding with vent location (Opatkiewicz et al., 2009; Huber et al., 2010; Makita et al., 2016). Although there were OTUs with significantly different abundances among all three groups of iron-dominated mats as determined via Metastats (Figure 3), there were no genera unique to one grouping, which suggests that a common ecosystem function is shared among the abundant community members of the Zeta-, Epsilon-, and Gamma-proteobacterial OTUs in all iron-dominated mats of the Mariana Arc and back-arc. This indicates that local fluid geochemistry (e.g., sulfur- vs. iron-dominated) rather than large-scale geography is more influential in determining the bacterial community composition and function, although there are observable patterns in biogeography based on differential OTU abundance among sites.

### Zetaproteobacteria

All characterized Zetaproteobacteria strains are obligate iron-oxidizing lithoautotrophs belonging to the genus *Mariprofundus* (Emerson et al., 2007; McBeth et al., 2011; Makita et al., 2017). Members of this genus have been previously detected in flocculent mats from the back-arc Snail and Urashima sites and from NW Eifuku (Davis and Moyer, 2008; Kato et al., 2009a; Makita et al., 2016); in addition to these locations, we have now identified Zetaproteobacteria at NW Rota-1 (Olde Iron Slides). After years of documented eruptions from 2004 to 2010 (Schnur et al., 2017), it is possible the hydrothermal fluids are undergoing a transition from sulfur- to iron-dominated effluent at this location as hypothesized by Butterfield et al. (1997). This geochemical succession is likely mirrored by the change in community structure that we are observing here as Zetaproteobacteria colonize a previously Epsilonproteobacteria-dominated vent field. We hypothesize a more recent transition from high sulfur to iron conditions might also explain why the orange mats at Olde Iron Slides were thinner and patchier in comparison to those found at Snail, Urashima, and Yellow Cone (Figure 1).

Because all Zetaproteobacteria isolates belong to the same genus, RDP classifies each Zetaproteobacterial sequence as identical; however, this underrepresents the actual diversity of this class. To further resolve the taxonomic diversity of the Zetaproteobacteria, McAllister et al. (2011) focused on their biogeography throughout the Pacific Ocean and found zOTUs 1 and 2 to be globally distributed, or cosmopolitan. In our study, zOTU 1 was found in relatively high abundance in all iron-dominated mats (Figure 3); however, zOTU 2 does not appear to be ecologically relevant or play a large role in ecosystem function, though it was detected throughout at low levels. Single cell amplified genomes from Lō'ihi Seamount show greater genetic diversity in zOTU 1 than zOTU 2 (Field et al., 2015). This higher genetic diversity may result in more phenotypic plasticity in zOTU 1, providing an advantage in the Mariana hydrothermal systems, where vent habitats are more heterogeneous (in terms of temperature and chemical composition) than locations such as Lō'ihi Seamount, where zOTU 2 is more abundant (McAllister et al., 2011; Field et al., 2015; Fullerton et al., 2017). The presence of zOTUs 3 and 4 in the Mariana raises their status to cosmopolitan across the Pacific. The higher depth of sequencing obtained here is probably responsible for their detection rather than a recent colonization event (although these are not mutually exclusive). The type strains *Mariprofundus ferrooxidans* and *Mariprofundus micogutta* belong to zOTUs 11 and 18, respectively. Though detected, these zOTUs were not found to be abundant in any mat communities investigated (Figure 3). Therefore, the present type strains are likely poor representatives of the most ecologically important and ubiquitously distributed zOTUs in the Mariana Arc and back-arc microbial mat communities.

The diversity of the Zetaproteobacteria has been under investigation due to their prominence and ecological importance in iron-rich hydrothermal habitats. Evidence suggests that Zetaproteobacteria not only act as primary producers (with respect to carbon cycling), but also produce an extensive physical environment that would otherwise not exist, thereby providing a habitat for diverse microbial communities. In a comparison between the two community types, all the Zetaproteobacterial-dominated mats had higher microbial diversity than the Epsilonproteobacteria-dominated community types (Table 3). This increased biodiversity is potentially due to the Fe-oxyhydroxides produced by the Zetaproteobacteria. These extracellular structures (e.g., stalks and sheaths) are well-documented from both isolates and naturally occurring microbial mats (Fleming et al., 2013; Bennett et al., 2014; Chan et al., 2016b; Makita et al., 2016). This high surface-area architecture can be colonized by an array of secondary consumers (e.g., Bacteroidetes, Chloroflexi, Gammaproteobacteria, and Planctomycetes) that were also found to be present (Figure 2).

### Epsilonproteobacteria

The Epsilonproteobacteria have broad metabolic capabilities, at hydrothermal vents they are typically represented by lithoautotrophic isolates energetically using reduced sulfur compounds and/or H_2_, they are motile and capable of quorum-sensing (Takai et al., 2005a; Campbell et al., 2006; Pérez-Rodríguez et al., 2015; Waite et al., 2017). Epsilonproteobacteria were detected in all samples; however, there are two distinct types of Epsilonproteobacterial communities containing OTUs either from the genera *Thioreductor* and *Lebetimonas* or *Sulfurovum* and *Sulfurimonas*. The sulfur-rich fluids at Champagne Vent were also high in H_2_ and supported a Thioreductor/Lebetimonas-dominated microbial mat. This was the only mat community composed of a high percentage of *Thioreductor* (Figure 3), which is represented by the type strain *Thioreductor micantisoli* (Nakagawa et al., 2005a). Cultured representatives in this genus are mesophilic, strictly anaerobic, and utilize H_2_ as their electron donor and S^0^ or ${\text{NO}}_{3}^{-}$ as electron acceptors (Nakagawa et al., 2005b). Champagne Vent was also the only mat community that contained a high percentage of reads classified as Lebetimonas (Figure 3). The type strain for this group is *Lebetimonas acidophila*, also a strictly anaerobic H_2_-oxidizer and S^0^-reducer (Takai et al., 2005b). Previous work found both Thioreductor and Lebetimonas sequences in hydrothermal fluids sampled from NW Eifuku and NW Rota-1 as well as other Mariana Arc and back-arc vent fields (Huber et al., 2010; Meyer and Huber, 2014). These two genera appear to be highly prevalent in fluids, but as shown here are often rare in microbial mats as abundances of Thioreductor/Lebetimonas OTUs were <0.05% in all mats other than at Champagne Vent. This is likely due to their metabolic requirement for H_2_, which strongly correlates with the concentration of H_2_ (Figure 4). The low diversity and evenness in the Champagne Vent mat (Table 3) was due to the dominance of four putative H_2_-oxidizing OTUs accounting for 71.5% of the total reads. This indicates that H_2_ was likely the primary energy source for this microbial mat community.

Epsilonproteobacteria found in the diffuse-flow, sulfur-dominated mat at Iceberg Vent contained abundant OTUs identified as Sulfurimonas and Sulfurovum. Type strain *Sulfurimonas autotrophica* is a strict aerobe oxidizing H_2_S, S^0^, and S_2_${\text{O}}_{3}^{2-}$ (Inagaki et al., 2004). *Sulfurovum lithotrophicum* also oxidizes S^0^ and S_2_${\text{O}}_{3}^{2-}$ aerobically or anaerobically with ${\text{NO}}_{3}^{-}$ as a terminal electron acceptor (Inagaki et al., 2003). We also detected a high abundance of Sulfurovum and Sulfurimonas OTUs in Zetaproteobacterial-dominated mats in addition to iron-oxidizing Zetaproteobacteria. Despite the low concentrations of H_2_S in iron-dominated mats, the Epsilonproteobacteria likely play an important role in biogeochemical cycling of sulfur and carbon in iron mats, and their ecological importance should not be overlooked.

### Gammaproteobacteria

The Gammaproteobacteria were also present in high abundance in mats with iron-dominated vent fluids, but the predicted metabolic potential is difficult to determine for the majority of them due to lack of classification beyond the class level. Six abundant Gammaproteobacterial OTUs classified as Methylococcaceae most likely obtain energy and carbon from methane (Hanson and Hanson, 1996; Gulledge et al., 2001). Methanotrophic Gammaproteobacteria in the order Methylococcaceae have been detected on the Mariana back-arc and other hydrothermal systems previously (Brazelton et al., 2006; Kato et al., 2009a). The other classified OTU belongs to the heterotrophic genus Thalassomonas (Macián et al., 2001). The remaining unclassified Gammaproteobacterial OTUs are likely common at marine hydrothermal habitats as indicated by high similarity to environmental sequences obtained from hydrothermal vents (Supplemental Table 1). When these same OTUs are compared to isolated strains the top representatives are heterotrophic or have sulfur-oxidizing metabolisms (Supplemental Table 1).

Globally, vent systems are rich in chemolithotrophic sulfur-oxidizing Gammaproteobacteria including free-living and invertebrate endosymbiont taxa (Wirsen et al., 1998; Reed et al., 2014). Known sulfur-oxidizing Gammaproteobacteria were present in low abundances in the mats in this study; e.g., six Thiomicrospira OTUs were detected, but the most abundant of these was only 0.18% of the total reads in sample 797B3 from Urashima. Recently, it has been shown that some *Thiomicrospira* spp. are also capable of switching between iron and sulfur oxidation (Barco et al., 2017). This metabolic switching could potentially cause some of the Gammaproteobacteria OTUs to more tightly cluster with those representing Zetaproteobacteria (Figure 4). Still, we hypothesize the abundant unclassified Gammaproteobacterial OTUs are predominantly heterotrophic (i.e., secondary consumers) in these Mariana, Zetaproteobacterial-dominated mats due to ecosystem engineering. The Gammaproteobacterial OTUs generally do not share a similar distribution pattern with the Sulfurovum/Sulfurimonas or Thioreductor/Lebetimonas OTUs, which suggests they do not have an energetic dependence on H_2_S or H_2_, respectively; however, there were two Gammaproteobacterial OTUs (055 and 153) that did share a similar distribution pattern with the Sulfurovum/Sulfurimonas, indicating that they may also share a potential energetic dependence on H_2_S (Figure 4). Making assumptions about the role of the unclassified Gammaproteobacteria in these habitats, however, must be done with caution because of the wide array of energy yielding metabolisms utilized by this phylogenetically diverse class (Williams et al., 2010).

### Sampling scale

In addition to a large geographic scale, we also compared communities of fine-scale biomat samples and bulk scoop samples taken from the same microbial mat on three occasions. Rarefaction analysis showed higher OTU richness in the scoop sample than in the corresponding biomat sample from all three mat communities (Supplemental Figure 1). Observation of the high abundance OTUs in these sample pairings revealed different OTU enrichments based on sample type (Supplemental Figure 2). The scoop and biomat pairing from NW Rota-1, Olde Iron Slides (800Sc8 and 800B12456) is a clear example where targeted, fine-scale sampling had a significant impact on the measured microbial diversity and community structure. The biomat sampler specifically targeted thin, orange tufts on the surface of the mat, and the scoop sampler was less selective and collected underlying sediment as well as the orange and white microbial mats. Correspondingly, the biomat sample had over twice as many Zetaproteobacterial reads (Figure 2) and was enriched in zOTUs 1 and 8 (Supplemental Figure 2). The higher diversity and richness exhibited in scoops (Table 3, Supplemental Figure 1) from the other two microbial mats (Snap-Snap, Urashima and Yellow Cone, NW Eifuku) is likely a result of sampling multiple microhabitats within these mats, e.g., oxygenated surface layers and microaerophilic to anoxic zones deeper in the architecture of the mat (Chan et al., 2016b). These data are similar to that of more accessible systems such as in photosynthetic microbial mats, where depth profiles have shown considerable changes in community structure at the millimeter scale (Harris et al., 2013). Sampling scale affected estimated bacterial diversity and should be considered in future assessments of hydrothermal microbial mat communities.

## Conclusions

This study offers insights into the community structure and biodiversity of lithotrophically-driven bacterial mat communities from iron- and sulfur-rich hydrothermal venting along the Mariana Arc and back-arc. Although local geochemistry (e.g., ratio of Fe/H_2_S and availability of H_2_) was the primary driver that correlated with community structure, geographic patterns in OTU abundance were also apparent, especially in the iron-dominated systems. Our study indicates an important functional role of Zetaproteobacteria at all sites with iron-dominated vent effluent and Epsilonproteobacteria at sites with sulfur-dominated fluids. Gammaproteobacteria were also high in abundance within the iron-dominated mats and likely had a heterotrophic role as secondary consumers, though some show the potential to grow lithotrophically as well. Higher bacterial diversity was observed in Zetaproteobacterial-dominated mats, which supports the hypothesis that Zetaproteobacteria function as ecosystem engineers altering the mat habitat and enhancing community interactions and complexity. In addition, sampling technique is an important consideration when attempting to assess the spatial heterogeneity associated with hydrothermal microbial mat communities. The high diversity observed among and within microbial communities encourages further research into the ecology, metabolic potential, and biodiversity of microbial mats fueled by Mariana Arc and back-arc submarine volcanism.

## Author contributions

This work was designed and samples were collected by KH, HF, and CM. Corresponding geochemical assays were designed and conducted by DB. KH conducted the molecular analyses and amplicon sequencing. KH, HF, and CM contributed to the bioinformatics. KH wrote the manuscript. HF, DB, and CM substantially contributed to the data interpretation, drafting and revising of the manuscript. KH, HF, DB, and CM are all responsible for all aspects of this work and approve the final version to be published.

### Conflict of interest statement

The authors declare that the research was conducted in the absence of any commercial or financial relationships that could be construed as a potential conflict of interest.
