# Supplementary figures and images for: Community Structure of Lithotrophically-Driven Hydrothermal Microbial Mats from the Mariana Arc and Back-Arc
Source: Front Microbiol. 2017 Aug 28;8:1578. doi: 10.3389/fmicb.2017.01578 (PMC5609546; doi:10.3389/fmicb.2017.01578)

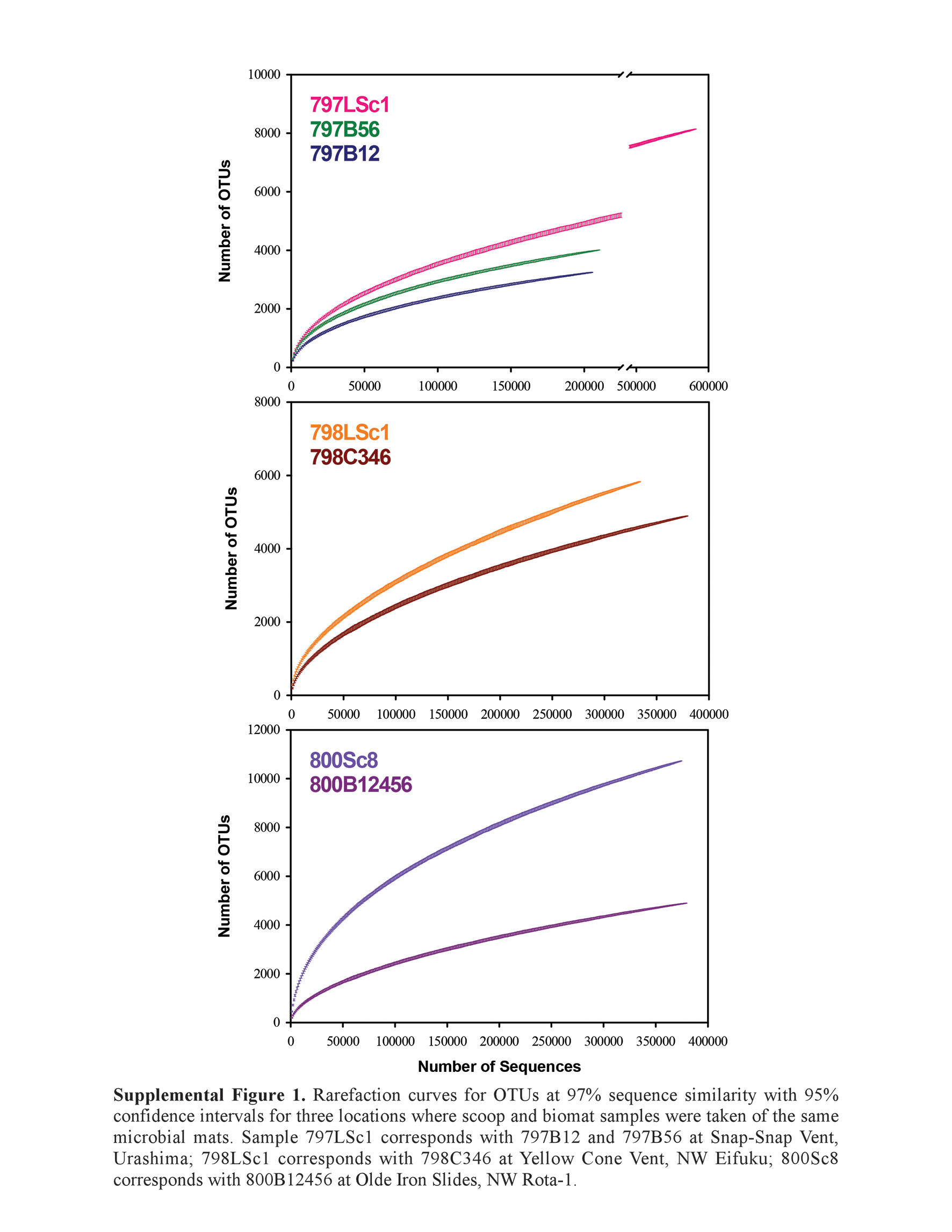

Supplement: Supplementary file 2 [file Image1.TIF]

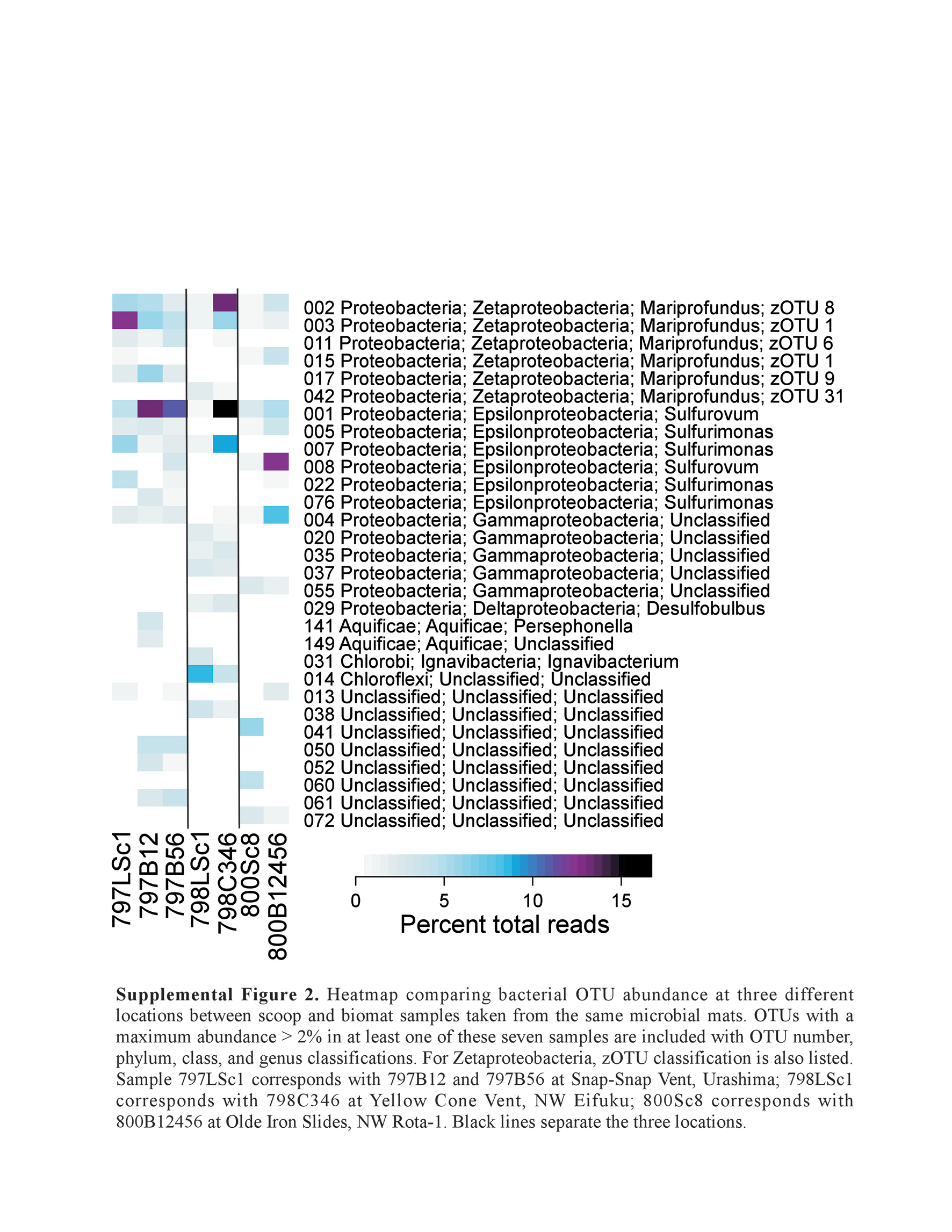

Supplement: Supplementary file 3 [file Image2.TIF]
